# Truncating the i-leader open reading frame enhances release of human adenovirus type 5 in glioma cells

**DOI:** 10.1186/1743-422X-8-162

**Published:** 2011-04-11

**Authors:** Sanne K van den Hengel, Jeroen de Vrij, Taco G Uil, Martine L Lamfers, Peter AE Sillevis Smitt, Rob C Hoeben

**Affiliations:** 1 Department of Molecular Cell Biology, Leiden University Medical Center, P.O. Box 9600, 2300 RC, Leiden, The Netherlands; 2 Department of Neuro-Oncology, Erasmus Medical Center, P. O. Box 5201, 3008 AE Rotterdam, The Netherlands

**Keywords:** glioma, gene therapy, adenovirus, i-leader, oncolytic virus

## Abstract

**Background:**

The survival of glioma patients with the current treatments is poor. Early clinical trails with replicating adenoviruses demonstrated the feasibility and safety of the use of adenoviruses as oncolytic agents. Antitumor efficacy has been moderate due to inefficient virus replication and spread. Previous studies have shown that truncation of the adenovirus i-leader open reading frame enhanced cytopathic activity of HAdV-5 in several tumor cell lines. Here we report the effect of an i-leader mutation on the cytopathic activity in glioma cell lines and in primary high-grade glioma cell cultures.

**Results:**

A mutation truncating the i-leader open reading frame was created in a molecular clone of replication-competent wild-type HAdV-5 by site-directed mutagenesis. We analyzed the cytopathic activity of this RL-07 mutant virus. A cell-viability assay showed increased cytopathic activity of the RL-07 mutant virus on U251 and SNB19 glioma cell lines. The plaque sizes of RL-07 on U251 monolayers were seven times larger than those of isogenic control viruses. Similarly, the cytopathic activity of the RL-07 viruses was strongly increased in six primary high-grade glioma cell cultures. In glioma cell lines the RL-07 virus was found to be released earlier into the culture medium. This was not due to enhanced viral protein synthesis, as was evident from equivalent E1A, Fiber and Adenovirus Death Protein amounts, nor to higher virus yields.

**Conclusion:**

The cytopathic activity of replicating adenovirus in glioblastoma cells is increased by truncating the i-leader open reading frame. Such mutations may help enhancing the antitumor cytopathic efficacy of oncolytic adenoviruses in the treatment of glioblastoma.

## Background

The poor prognosis of high grade gliomas with the current treatments prompted an ongoing search for alternative treatments. A new strategy for glioma treatment involves the use of viruses as oncolytic agents, such as Human Adenoviruses (HAdV), Herpes Simplex viruses and, more recently Reoviruses [1-5]. Of these, the use of HAdV has been explored most rigorously, including replication-defective HAdV vectors carrying heterologous transgenes, as well as replication-competent HAdV in which replication is restricted to tumor cells.

HAdV transduce both dividing and quiescent cells with high efficiency, they can be genetically modified with relative ease, and the technology for clinical-grade production is available. Their biology, which is understood in detail, also facilitates modification of the viral genome for creating Conditionally-Replicating Adenoviruses (CRAds). In addition, viral-tissue tropism can be modified by incorporating ligands that target specific receptors on tumor cells, for example by fusing ligands with the fiber [6] or with the minor capsid protein IX [7]. Also modifications have been described that promote interactions with the tumor-specific receptors. Such mutations can be used to increase transduction of target tissues [8-11].

The first phase I clinical trial with a replication-competent HAdV on malignant glioma was performed with ONYX-015 [1], which is based on HAdV-5 and harbors a deletion in the open reading frame encoding the 55 kDa E1B protein [12]. Although ONYX-015 has anti-tumor activity, the precise mechanism behind its tumor-cell preference is still controversial [13]. While the ONYX-015 study provided evidence of the safety of CRAds in glioma-patients [1], the anti-tumor efficacy of this virus was limited, presumably due to inefficient replication and poor intratumoral spread.

To isolate HAdV-5 mutants with improved cytopathic activity, two groups used random mutagenesis and bioselection strategies. Both studies yielded mutants containing point mutations in the i-leader region of the late transcription unit [14,15]. The i-leader is a 440-nucleotide long sequence that is found between the 2^nd ^and 3^rd ^element of the tripartite-leader sequence in a significant fraction of the major-late transcripts. This sequence contains an open reading frame which encodes a small protein of approximately 16 kDa in size [16]. It has been suggested that it reduces the half-life of L1 mRNAs, however the precise function of the i-leader protein is unknown [16,17]. A common point mutation, C8350T, which created a stop codon in the i-leader open reading frame, was isolated by Yan et al [15] after bioselection on the HT29 colon carcinoma cell line. Also the C8299T mutation, isolated by Subramanian and coworkers in a screen for large plaques on A549 adenocarcinomic human alveolar basal epithelial cells, truncated the i-leader open reading frame [14]. Yan and colleagues demonstrated that their mutants ONYX-201 and ONYX-203, which contain next to the i-leader mutation some additional mutations, strongly enhanced the cytopathic activity of HAdV-5 not only in the cells used for bioselection, but in a larger panel of tumor cell lines [15]. Unfortunately, no glioma cell lines were included in their study.

In this study we compared the cytopathic activity of a HAdV-5 virus containing the single i-leader point mutation with that of wtHAdV-5 in glioma cultures. To do so, we created a HAdV-5 that contains the C8350T mutation, which truncates this open reading frame. To facilitate identification of the mutant-virus genomes, an XhoI restriction site was created 6 nucleotides downstream of this mutation. We found the cytopathic activity in glioma cells of the mutant vector (RL-07) to be strongly increased compared to the wild-type vector (wtHAdV-5). While the mutation does not affect timing of viral protein synthesis and virus yields, the mutant virus was found to be released earlier from infected cells. These results demonstrate that improved adenoviruses with enhanced spreading activity in glioma cells can be generated by introduction of a mutation that truncates the i-leader. Such mutations may help enhancing the antitumor efficacy of oncolytic adenoviruses in the treatment of gliomas.

## Materials and methods

### Cell lines

In this study we used the malignant glioma cell lines U251 and SNB19, the HAdV-5 E1 transformed human embryonic retinoblast cell lines 911 and PER.C6, and the A549 lung carcinoma cell line as described before [18]. Cultures of freshly resected glioma cells were started from resection material of the Dept of Neurosurgery at Erasmus MC and used before the fifth passage. All cell lines were cultured in Dulbecco's modified Eagle's medium (DMEM, Gibco-BRL, Breda, the Netherlands) supplemented with 8% fetal bovine serum (FBS, Gibco-BRL) and penicillin-streptomycin at 37°C in a humidified atmosphere containing 5% CO_2_.

### Adenovirus vector

We used replication-competent HAdV-5 vectors. The mutant, containing the i-leader mutation C8350T and the extra XhoI restriction site (HAdV-5/RL-07), was generated by homologues recombination in E. coli BJ5183, using the pTG3602 plasmid [19] as backbone. The mutated i-leader fragment was generated by mutagenesis PCR using synthetic oligo nucleotides 5'- GACAACATCGCG**T**AGATGA*CTCGAG*TCCATGGTCTGG and 3'- CCAGACCATGGA*CTCGAG*TCATCT**A**CGCGATGTTGTC. The nucleotide corresponding to nt 8350 is represented in bold, and the XhoI restriction site is italicized and underlined. The HAdV-5/RL-07 virus was rescued on 911 cells, propagated on PER.C6 cells, purified by double CsCl density gradient centrifugation, dialyzed, and stored at -80°C in sucrose buffer. Infectious particle titers were determined by plaque assay on 911 cells. The presence of the C8350T mutation and the XhoI restriction site was verified by sequence analyses (*supplementary data*). The wtHAdV-5 virus used in this study was recovered in identical manner, using a wt HAdV-5 i-leader fragment, to ensure isogenicity of the RL-07 virus and the wtHAdV-5. The nucleotide sequence of the entire RL-07 virus is available upon request.

### Viral titer

Plaque assays were performed to determine the biological titers (as plaque forming units per ml) as described [20]. Briefly, 911 cells were seeded in 6-well plates 1 day prior to viral infection. The cultures were exposed to serial dilutions of the virus. After 4 hours viral medium was removed and cells were overlaid with 0.5% agar/F15 media supplemented with 2% horse serum. Plaque titers were read at day 10 post infection (p.i.).

The concentrations of viral genome copies were determined by picogreen assay [21]. Purified virus was inactivated in a final concentration of 0.05% SDS in storage buffer and loaded in microtiter plates. After adding picogreen, the fluorescence was measured and viral genomes were calculated using purified bacteriophage λ DNA as a standard.

### Propagation on 911 cells

911 cells were seeded in 6-well plates 1 day before infection. Cultures were infected with wtHAdV-5 or RL-07 in parallel MOI = 3. 48 hrs p.i. virus was harvested and viral titers were defined by plaque assays as described above.

### Cell viability assay

WST-1 reagent (Roche, Woerden, The Netherlands) was used to determine the viability of the cultures after adenoviral infection. Cells were seeded in 96-well plates and infected with various amounts of virus. The cell viability of the cultures was determined 4 days p.i. for the established cell lines, and 7 days p.i. for the primary glioma cultures by adding the WST-1 reagent according to the manufacturer's instructions.

### Viral plaque formation

The plaque surfaces were determined on U251 cells according the plaque titration protocol as for 911 cells. The agar was removed 10 days p.i., and the cells were subsequently fixed with methanol and stained with 0.5% crystal violet solution. The relative plaque surfaces were determined with Olympus DP-software.

### Viral release

To determine the viral release, media and cells were collected separately at 30 and 48 hrs p.i. of U251 and SNB19 cultures infected with wtHAdV-5 and RL-07 at MOI = 3. The titers were determined by plaque titration on 911 cells.

### Immunoblot procedure

To study the viral protein synthesis after infection an immunoblot procedure was used. U251 and SNB19 were seeded in 6-well plate o/n and infected with RL-07 or HAdV-5 at MOI = 1. Cells were harvested 24, 40, 48, 64, and 70 hrs p.i. in radioimmunoprecipitation assay lysis buffer (Ripa) (50 mM Tris-Cl, pH 7.5, 150 mM NaCl, 0.1% SDS, 0.5% DOC, and 1% NP40) supplemented with protease inhibitors (Complete mini tablets, Roche Diagnostics, Almere, The Netherlands). Per sample 50 μg of protein lysate, defined by the Pierce BCA protein assay kit (Thermo Scientific Rockford, USA), and after addition of western sample buffer (final concentrations: 10% glycerol, 2% SDS, 50 mM Tris-Cl (pH 6.8), 2.5% β-mercaptoethanol and 0.025% bromophenol blue) was loaded onto a SDS-polyacrylamide gel. The proteins were transferred to Immobilon-P (Millipore, Etten-Leur, The Netherlands). Detection of fiber, E1A and ADP was performed with the antibodies 4D2 (anti-fiber tail [22]) and M73 (anti-E1A [23]), a rabbit antiserum raised against an ADP peptide (P63-77 [24]; a kind gift from Dr. W. S. M. Wold), respectively, and visualized by enhanced chemiluminescence. ImmunO anti-actin clone C4 (MP Biomedicals, Amsterdam, The Netherlands) was used as loading control.

### Colony-based survival assay

Suspensions of U251 and SNB19 were infected with wtHAdV-5 or RL-07 with a MOI = 100 in 2%FBS/DMEM, non-infected cells were used as control. Cells were incubated for 1 hr at 37°C, washed 3 times with PBS supplemented with 1:500 diluted Immunoglobluline I.V. (stock 60 mg/ml) and plated in a concentration of 500 cells/dish, medium was supplemented with 1:500 dilution of IVIG. After 10 days cells were fixed with methanol and stained with 0.5% crystal violet solution. Single colonies were counted and number of colonies of wtHAdV-5 was compared to the number RL-07 colonies.

### Trypan blue exclusion-based survival assay

This procedure was performed as described by Tollefson et al [25]. In short, U251 and SNB19 were seeded at 4.10^5 ^cells per well in a 6-well plate. The next morning cells were infected with MOI = 100 in 1 ml DMEM/2%FBS. After 2 hr 1 ml DMEM/14%FBS was added to each well. From day 1 up to day 5 media and cells were harvested and trypan blue was added to the cells in a final concentration of 0.02%. A total of 500-600 cells were counted and percentage of viable cells was calculated.

## Results

The aim of this study was to evaluate whether truncation of the i-leader open reading frame enhances the cytopathic effect of HAdV-5 in human glioma cell lines. Since the ONYX-234 virus [15], which consists of HAdV-5 with the C8350T mutation, was unavailable to us, we recreated this mutant by site-directed mutagenesis and homologous recombination. To facilitate straightforward identification of this mutant, we inserted an XhoI-restriction site 6 nucleotides downstream of the C8350T mutation (additional files 1 and 2). From the cloned virus DNA, viruses were generated by transfection of the viral genomes on 911 helper cells. Viral plaques were picked and the viruses expanded and propagated on PER.C6 cells. No consistent differences in viral particle to plaque forming unit (pfu) ratios were observed (table 1) in RL-07 and wtHAdV-5 virus bathes produced in parallel. Also on 911 cells, the RL-07 viruses could be propagated efficiently, with the kinetics and yields very similar to the wtHAdV-5 (table 2).

**Table 1:** Overview viral yield in pfu/ml and vp/ml after CsCl-purification

		*Plaque assay*	*Picogreen assay*	*vp/pfu ratio*
**Batch 1**	***wtHAdV-5***	3 × 10^11 ^pfu/ml	5.5 × 10^12 ^vp/ml	18
	***RL-07***	1.1 × 10^11 ^pfu/ml	1.6 × 10^12 ^vp/ml	15

**Batch 2**	***wtHAdV-5***	2.8 × 10^11 ^pfu/ml	3.2 × 10^12 ^vp/ml	11
	***RL-07***	1.3 × 10^11 ^pfu/ml	1.6 × 10^12 ^vp/ml	12

**Table 2:** Viral yield (pfu) of wtHAdV-5 and RL-07 on 911 cultures

	wtHAdV-5	RL-07
culture 1	1.35E+09	1.24E+09
culture 2	1.42E+09	1.13E+09
culture 3	1.25E+09	1.34E+09

**Average**	**1.34E+09**	**1.23E+09**
***SD***	***8.81E+07***	***1.08E+08***

**Figure 1:**
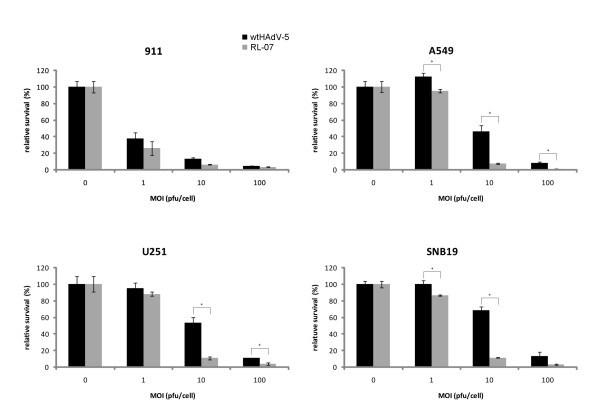
**Relative survival as determined by WST-1 assay**. Cytopathic activity of RL-07 (grey bar) compared to wtHAdV-5 (black bar) is higher on glioma cell lines U251 and SNB19. Error bars represent the SD (n = 3). * - statistical significance; unpaired, two-sided t-test, p < 0.01. Cells were seeded in 96-well plates 1 day before infection with RL-07 and wtHAd5 (MOI range 0-100 pfu/cell). Viability of the cultures was determined 4 days p.i by WST-1 assay. Very similar results were obtained in a repeat experiment.

To analyze the cytopathic activity of RL-07, two glioma cell lines (U251 and SNB19, both CAR-positive) and two control cell lines (911 and A549) were infected with RL-07 and wtHAdV-5 at multiplicity of infections (MOI) ranging from 0 to 100 pfu/cell. Four days p.i., the cell viability was determined by WST-1 assay (Figure 1), RL-07 had a significant higher cytopathic effect on glioma cell lines and A549 compared to the control virus wtHAdV-5. On 911 cells, no differences in the induction of cytopathic effects were detected.

**Figure 2:**
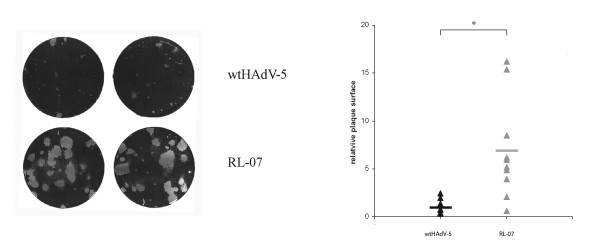
**Viral plaque formation on U251 cells**. Monolayers of U251 were infected with RL-07 or wtHAdV-5 with a MOI of 2.2 × 10^-5 ^and 6 × 10^-5 ^respectively, and overlaid with 0.5% agar in F15 media supplemented with 2% Horse serum. Ten days p.i. the agar was removed and cells were stained with 0.5% crystal violet solution. (A) Photograph of plaques of wtHAdV-5 and RL-07 on U251 monolayer (B) Relative plaque surface of 10 plaques. *-statistical significance; unpaired, two-sided, t-test, p < 0.01. wtHAdV-5 - black closed triangles; RL-07 - grey closed triangles.

To determine whether the decreased viability of RL-07 infected cells in the WST-1 assay correlates with enhanced spread in monolayers of glioma cells, a plaque assay was performed on U251 cells. Monolayers of U251 were infected with RL-07 and wtHAdV-5 with a 10^9 ^times diluted virus stock (batch 1) and overlaid with agar. Plaque sizes were scored 10 days p.i. (Figure 2). The plaques formed upon infection with RL-07 are on average 7-fold larger in size than the plaques obtained with wtHAdV-5, demonstrating that the mutation enhances viral spread.

**Figure 3:**
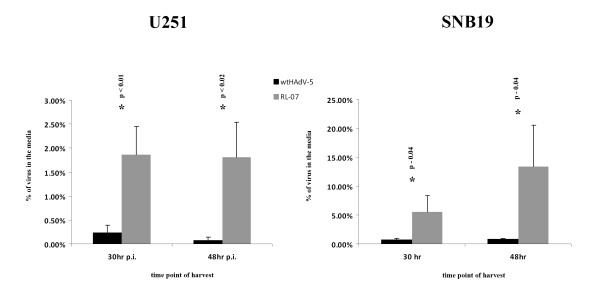
**Virus releases in culture media**. At 30 and 48 hrs p.i. with wtHAdV-5 and RL-07 the cells and the media of the U251 and SNB19 cultures were collected separately and assayed in a plaque assay. The fraction of the total yield recovered in the medium is plotted. Black bars represent wtHAdV-5 and the grey bars RL-07. Error bars represent the SD (n = 3)* - *statistical significance; unpaired, two sided t-test*.

To compare the kinetics of virus release, U251 and SNB19 cells were infected with either RL-07 or wtHAdV-5. At 30 and 48 hours p.i. the medium was carefully separated from the adherent cells and both were collected. In both fractions the viral particles were quantified by plaque assays on 911 cells (Figure 3). In both cell lines approximately 7-fold more virus was found in the medium of the RL-07 infected cells than in the medium of the wtHAdV-5 infected cells at 30 hrs p.i.. At 48 hrs p.i. media of RL-07 infected U251 cells contained approximately 22 times more virus than those infected with wtHAdV-5. Similarly, the amount of RL-07 in the media of SNB19 cultures was at least 13 times higher at 48 hrs p.i.. From these data we conclude that the RL-07 virus is released in to the medium earlier than wtHAdV-5.

**Figure 4:**
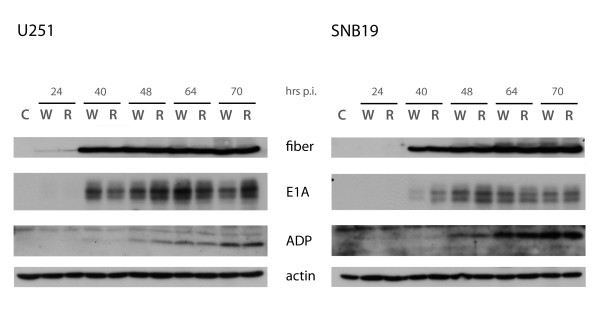
**Protein immunoblot assay for detection of viral proteins**. Fiber, E1A, ADP levels were detected upon infection with RL-07 or wtHAdV-5 at various time points p.i.. Actin was used as loading control. Cells were seeded o/n in a 6-well plate and infected with MOI = 1. Cells were harvested 24, 40, 48, 64 and 70 hrs p.i. in Ripa buffer. Protein amounts were determined by BCA protein assay. In each lane 50 μg of protein was loaded. C-mock infected control; W- infected with wtHAdV-5; R- infected with RL-07.

Having established that the RL-07 virus was released earlier than wtHAdV-5, we examined the expression pattern of the viral proteins E1A, fiber, and the adenovirus death protein (ADP). U251 and SNB19 cells were infected at MOI = 1 and harvested at several time points after infection and viral proteins were visualized by immunoblotting (Figure 4). In our time series no differences between fiber, E1A and ADP levels were observed between RL-07 and wtHAdV-5. This shows that the rapid release of RL-07 particles does not result from more rapid viral protein synthesis.

**Figure 5:**
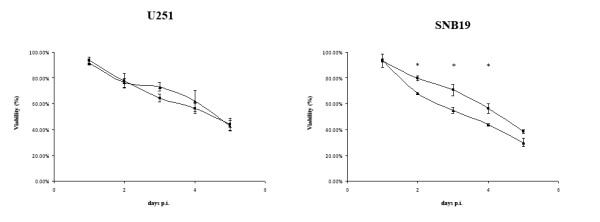
**Trypan blue-based survival assay**. U251 and SNB19 cultures were infected with wtHAdV-5 or RL-07 with a MOI of 100. Cell death in the cultures was determined by trypan blue as described by Tollefson and colleagues [25]. Error bars represent the SD (n = 3) * - *statistical significance; unpaired, two sided t-test, p < 0.01*. ♦ - wtHAdV-5 infected and ▲ - RL-07 infected.

In addition to the WST-1 assay, which determines the metabolic activity of the remaining viable cell population, two more direct survival assays were performed. These are the colony-based survival assay and the trypan blue exclusion-based survival assay. The colony-based survival assay measures the number of residual colony-forming cells after exposure of the cells to the virus. In this assay no differences in the number of colonies were noted between wtHAdV-5 and RL-07-infected U251 or SNB19 cells (data not shown). With the trypan-blue based assay the fraction of dead cell was assessed by trypan-blue, starting 1 day after infection with MOI = 100. Up to day 5 the cell viability of the U251 and SNB19 cultures were determined and plotted (Figure 5). At one day p.i. there was no increase in cell death discernable. At two days after infection the viability started to decrease. On the U251 cells no differences were observed between the two viruses. On the SNB19 cell line, from day 2 up to day 5 p.i. a significant higher proportion of dead cells were detected in the cultures infected with RL-07 compared to wtHAdV-5. This indicated that RL-07 accelerates cell death on this cell line.

**Figure 6:**
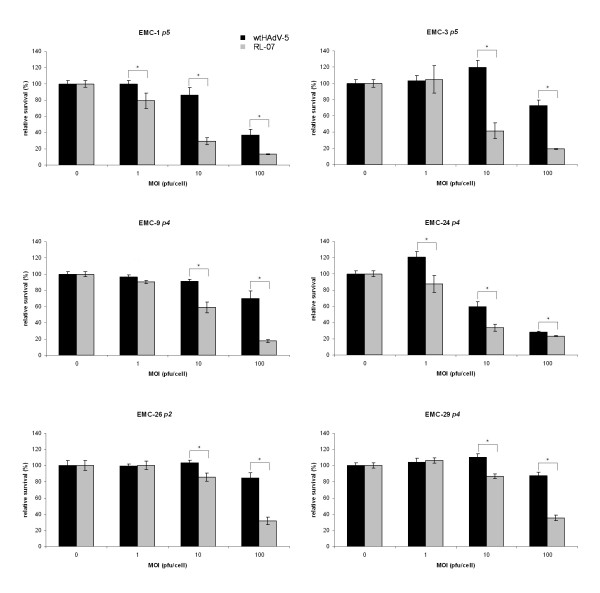
**Relative survival of infected cells as determined by WST-1 assay**. Cytopathic activity of RL-07 (grey bar) compared to wtHAdV-5 (black bar) is higher on primary high grade glioma cell cultures. Error bars represent SD (n = 4). * - statistical significance; paired, two sided t-test, p < 0.01. Cells were seeded in 96-well plates 1 day prior infection with RL-07 and wtHAdV-5 (MOI range 0-100 pfu/cell). Viability of the cultures was determined by WST-1 assay 7 days p.i..

So far our results demonstrate that the i-leader mutation in RL-07 accelerates virus release and enhances spread in established glioma cell lines. However, the potency of oncolytic HAdV can differ remarkably between established cell lines and cultures derived from resection material. To verify the cytopathic activity of RL-07 in human gliomas, cultures established from resection material of high grade gliomas were used for cytolysis assays. The characteristics of the cultures used in this study are summarized in Table 3. The cytopathic activities of the viruses were evaluated 7 days p.i. by WST-1 assay. The results are presented in Figure 6. At MOI = 10 and MOI = 100 all RL-07-infected cultures showed a significantly increased cytopathic activity in comparison to wtHAdV-5. In three of the cultures, EMC-1, EMC-9 and EMC-24, a significant effect could already observed at MOI = 1.

**Table 3:** Characteristics primary glioma cell cultures

Tumor specimen	Male/Female	Age	Histology
**EMC-1**	M	44	Anaplas. Astrocytoma
**EMC-3**	M	48	Glioblastoma multiform
**EMC-9**	M	66	Glioblastoma multiform
**EMC-24**	M	50	Glioblastoma multiform
**EMC-26**	F	42	Anaplas. Astrocytoma
**EMC-29**	F	53	Anaplas. Astrocytoma

## Discussion

In our long term efforts of developing efficacious adenoviruses for gene therapy in glioma [9,18,26-30], we studied the effect of a truncating mutation in the i-leader open reading frame on adenovirus cytopathic activity in glioma cell lines and cell cultures. Our data show that this mutation results in more rapid release of progeny viruses into the culture medium and enhanced spread of the virus in plaque assays. While the precise mechanism for this effect remains to be elucidated, the improved cytopathic activity may be employed in new oncolytic adenoviruses. Our data confirm and extend the results described by Yan et al [15]. These authors isolated the ONYX-201 and ONYX-203 viruses by bioselection on HT29 cells. These viruses harbor several mutations, and share four mutations including the i-leader truncating C8350T mutation. Subsequently these authors generated the ONYX-234 virus that only harbors the C8350T mutation, and show that this mutation enhances the adenovirus's capacity to kill HT29 cells. We confirm and extend these observations and demonstrate that truncation of the open reading frame enhances the adenovirus's cytopatogenicity in glioma cells.

Similar to Subramanian et al [14], we found that the i-leader mutation did not affect virus yield and production kinetics. In addition, no consistent differences in particle to pfu ratios were observed in batches produced in parallel. In a side by side comparison we demonstrated that RL-07 is more cytopathic on A549 and the glioma cell lines U251 and SNB19 than the isogenic wtHAdV-5. The increased cytopathic activity observed on these cell lines is similar to the observations of Yan et al with Onyx-234 on HT29 [15].

Enhanced spread was observed on U251 (Figure 2) with a 7-fold increase in plaque surface. This is in line with published data of the other i-leader mutants [14,15]. There is a broad variation in plaque surfaces for both viruses. As discussed by Subramanian et al this may be attributable to stochastic fluctuations in virus infection and replication [14]. The enhanced spread on U251 can be explained by the more rapid release of RL-07 from the cells. This is not caused by enhanced DNA replication (*data not shown*) confirming observations of Soloway and Shenk [17] and Subramanian [14], nor by a changed kinetics of protein synthesis of fiber, E1A and ADP (Figure 3). These results are similar to the results of Subramanian and co-workers, who also demonstrated that the improved viral spread is independent of ADP expression [14]. In contrast to the data of Yan et al [15], we found no evidence that the truncating i-leader mutation affects virus replication, viral gene expression, or viral yields. The reason for this discrepancy is unclear but may relate to differences in the cell system used (glioma cells vs. colorectal carcinoma cells). It is also conceivable that any of the other six mutations in the ONYX-201 and ONYX -203 viruses have contributed to the differences between the results of the Yan study and ours [15].

Our data provide clear evidence for enhanced cytopathicy of the RL-07 virus in glioma cell cultures. To test whether this is caused by more rapid cell death upon infection or to enhanced virus spread we used several assays to assess cell viability. The WST-1 assay measures the metabolic activity of the residual viable cells in a culture, and is unable to distinguish these two components. The involvement of ADP in early cell death is unlikely since there is no difference between ADP expression in RL-07 and HAdV-5 infected cells. A more direct viability assays is the colony-formation survival assay which yielded very similar results with RL-07 and wtHAdV-5. Also a more direct assessment of cell killing on a per-cell basis is the assay by trypan-blue exclusion. This assay revealed that there was no difference in cell death between the two viruses in U251 cells. On SNB19 cells a slightly more rapid cell death was observed in cultures infected with RL-07 compared to wtHAdV-5. The reason for the difference between the U251 cells and the SNB19 cells is unclear. Nevertheless, our data imply that although metabolic viability of U251 cultures infected with RL-07 decreases faster than in wtHAdV-5 infected cultures, this is not reflected in a more rapid cell killing by RL-07. In both cell types there is a strong increase in the release of infectious viral particles early after infection (Figure 3).

In the last set of experiments the cytopathic activity of RL-07 was determined on primary glioma cell cultures. Primary cell cultures resemble the original tumor more than established tumor cell lines. Also in these cultures, we observed significant increased cytotoxic activity of RL-07 in all the cell lines tested. These results confirm that RL-07 exhibits an increased cytopathic activity in low passage cultures of resected glioma cells.

To enhance the potency and reduce the toxicity of adenovirus vectors the replication of HAdV-5 vectors for glioma therapy has been restricted by using promoters that are preferentially expressed in tumor cells for driving expression of key viral genes such as E1A. Also the tropism of the viruses has been modified by fiber modifications to increase the transduction of tumor cells and/or to decrease the transduction in non-target cells. New mutations such at the mutation that truncates the i-leader open reading frame may prove very useful for optimizing such vectors by increasing their the spread. With the fiber-swap mutations [9] and the optimized glial fibrillary acidic protein promoter [18,28], the mutation truncating the i-leader open reading frame protein constitutes a new addition to our arsenal of validated modules for inclusion in new replicating glioma-targeted adenoviruses.

## Conclusion

In conclusion, truncating the i-leader of HAdV-5 by a single point mutation will give increased cytopathic activity in glioma cells. Introducing this mutation in an oncolytic adenovirus may enhance the antitumor cytopathic efficacy in the treatment of glioblastoma.

## Competing interests

The authors declare that they have no competing interests.

## Authors' contributions

SKvdH, MLL, PAESS, RCH designed research; SKvdH, JdV, TGU performed experiments; JdV, TGU, MLL, PAESS contributed vital new reagents and cells; SKvdH, JdV, RCH analyzed data; SKvdH, MLL, PAESS, RCH interpreted data. JdV, TGU, MLL, PAESS contributed unpublished experiments that helped the direction of the work; SKvdH, RCH wrote the manuscript.

## Supplementary Material

Additional file 1 **Identification RL-07**. Identification of RL-07 by PCR analyses. PCR analyses were performed on small freeze-thaw-lysate samples of the viral batches. Samples were heat inactivated and treated with proteinase K prior to PCR analysis. The i-leader region was amplified with the following primer set: Fwd primer 5'-AGACGCTCGGTGCGAGGATGCG; Rev primer 3'-GTCGTCTTCACGCAGAGGCGC. The PCR product was purified according to the SureClean protocol (Bioline, London, UK) and the product was digested using XhoI and loaded on 2% agarose gel. The picture represents the photo-negative image. Click here for file

Additional file 2 **Alignment i-leader region**. Sequencing analyses were performed on PCR products of freeze-thaw samples of virus batches (described by identification RL-07). PCR products were first cleaned with Sureclean (Bioline, London, UK), according to the manual, before direct sequencing. Sequencing was performed at the Leiden Genome Technology Center (LGTC, Leiden, The Netherlands). The sequences of nucleotide 8300-8400 of the parental plasmid pTG3602 and the sequences of the HAdV-5 and RL-07 viruses are represented. The C8350T changes, as well as the XhoI site created in the RL-07 virus, are boxed. Click here for file

## References

[B1] Chiocca EA Abbed KM Tatter S Louis DN Hochberg FH Barker F A phase I open-label, dose-escalation, multi-institutional trial of injection with an E1B-Attenuated adenovirus, ONYX-015, into the peritumoral region of recurrent malignant gliomas, in the adjuvant setting Mol Ther 2004 10 585 598 10.1016/j.ymthe.2004.07.021 15509513

[B2] Forsyth P Roldan G George D Wallace C Palmer CA Morris D A phase I trial of intratumoral administration of reovirus in patients with histologically confirmed recurrent malignant gliomas Mol Ther 2008 16 627 632 10.1038/sj.mt.6300403 18253152

[B3] Markert JM Medlock MD Rabkin SD Gillespie GY Todo T Hunter WD Conditionally replicating herpes simplex virus mutant, G207 for the treatment of malignant glioma: results of a phase I trial Gene Ther 2000 7 867 874 10.1038/sj.gt.3301205 10845725

[B4] Pulkkanen KJ Yla-Herttuala S Gene therapy for malignant glioma: current clinical status Mol Ther 2005 12 585 598 10.1016/j.ymthe.2005.07.357 16095972

[B5] Sonabend AM Ulasov IV Lesniak MS Conditionally replicative adenoviral vectors for malignant glioma Rev Med Virol 2006 16 99 115 10.1002/rmv.490 16416455

[B6] Glasgow JN Everts M Curiel DT Transductional targeting of adenovirus vectors for gene therapy Cancer Gene Ther 2006 13 830 844 10.1038/sj.cgt.7700928 16439993 PMC1781516

[B7] De Vrij J Uil TG van den Hengel SK Cramer SJ Koppers-Lalic D Verweij MC Adenovirus targeting to HLA-A1/MAGE-A1-positive tumor cells by fusing a single-chain T-cell receptor with minor capsid protein IX Gene Ther 2008 15 978 989 10.1038/gt.2008.26 18323790

[B8] Van Houdt WJ Wu H Glasgow JN Lamfers ML Dirven CM Gillespie GY Gene delivery into malignant glioma by infectivity-enhanced adenovirus: in vivo versus in vitro models Neuro Oncol 2007 9 280 290 10.1215/15228517-2007-017 17522331 PMC1907413

[B9] Brouwer E Havenga MJ Ophorst O de Leeuw B Gijsbers L Gillissen G Human adenovirus type 35 vector for gene therapy of brain cancer: improved transduction and bypass of pre-existing anti-vector immunity in cancer patients Cancer Gene Ther 2007 14 211 219 10.1038/sj.cgt.7701010 17082793

[B10] Nandi S Ulasov IV Rolle CE Han Y Lesniak MS A chimeric adenovirus with an Ad 3 fiber knob modification augments glioma virotherapy J Gene Med 2009 11 1005 1011 10.1002/jgm.1385 19688792 PMC2793323

[B11] Zheng S Ulasov IV Han Y Tyler MA Zhu ZB Lesniak MS Fiber-knob modifications enhance adenoviral tropism and gene transfer in malignant glioma J Gene Med 2007 9 151 160 10.1002/jgm.1008 17351980

[B12] Bischoff JR Kirn DH Williams A Heise C Horn S Muna M An adenovirus mutant that replicates selectively in p53-deficient human tumor cells Science 1996 274 373 376 10.1126/science.274.5286.373 8832876

[B13] Edwards SJ Dix BR Myers CJ Dobson-Le D Huschtscha L Hibma M Evidence that Replication of the Antitumor Adenovirus ONYX-015 Is Not Controlled by the p53 and p14ARF Tumor Suppressor Genes J Virol 2002 76 437 445 10.1128/JVI.76.24.12483-12490.2002 PMC136704 12438574

[B14] Subramanian T Vijayalingam S Chinnadurai G Genetic identification of adenovirus type 5 genes that influence viral spread J Virol 2006 80 2000 2012 10.1128/JVI.80.4.2000-2012.2006 16439556 PMC1367173

[B15] Yan W Kitzes G Dormishian F Hawkins L Sampson-Johannes A Watanabe J Developing novel oncolytic adenoviruses through bioselection J Virol 2003 77 2640 2650 10.1128/JVI.77.4.2640-2650.2003 12552003 PMC141112

[B16] Symington JS Lucher LA Brackmann KH Virtanen A Pettersson U Green M Biosynthesis of adenovirus type 2 i-leader protein J Virol 1986 57 848 856 3005631 10.1128/jvi.57.3.848-856.1986 PMC252814

[B17] Soloway PD Shenk T The adenovirus type 5 i-leader open reading frame functions in cis to reduce the half-life of L1 mRNAs J Virol 1990 64 551 558 2296076 10.1128/jvi.64.2.551-558.1990 PMC249143

[B18] de Leeuw B Su M ter Horst M Iwata S Rodijk M Hoeben RC Increased glia-specific transgene expression with glial fibrillary acidic protein promoters containing multiple enhancer elements J Neurosci Res 2006 83 744 753 10.1002/jnr.20776 16496373

[B19] Chartier C Degryse E Gantzer M Dieterle A Pavirani A Mehtali M Efficient generation of recombinant adenovirus vectors by homologous recombination in Escherichia coli J Virol 1996 70 4805 4810 8676512 10.1128/jvi.70.7.4805-4810.1996 PMC190422

[B20] Fallaux FJ Kranenburg O Cramer SJ Houweling A Van Ormondt H Hoeben RC Characterization of 911: a new helper cell line for the titration and propagation of early region 1-deleted adenoviral vectors Hum Gene Ther 1996 7 215 222 10.1089/hum.1996.7.2-215 8788172

[B21] Murakami P McCaman MT Quantitation of adenovirus DNA and virus particles with the PicoGreen fluorescent Dye Anal Biochem 1999 274 283 288 10.1006/abio.1999.4282 10527527

[B22] Hong JS Engler JA The amino terminus of the adenovirus fiber protein encodes the nuclear localization signal Virology 1991 185 758 767 10.1016/0042-6822(91)90547-O 1962447

[B23] Harlow E Franza BR Schley C Monoclonal antibodies specific for adenovirus early region 1A proteins: extensive heterogeneity in early region 1A products J Virol 1985 55 533 546 3894685 10.1128/jvi.55.3.533-546.1985 PMC255001

[B24] Tollefson AE Scaria A Saha SK Wold WS The 11,600-MW protein encoded by region E3 of adenovirus is expressed early but is greatly amplified at late stages of infection J Virol 1992 66 3633 3642 1316473 10.1128/jvi.66.6.3633-3642.1992 PMC241146

[B25] Tollefson AE Scaria A Hermiston TW Ryerse JS Wold LJ Wold WS The adenovirus death protein (E3-11.6K) is required at very late stages of infection for efficient cell lysis and release of adenovirus from infected cells J Virol 1996 70 2296 2306 8642656 10.1128/jvi.70.4.2296-2306.1996 PMC190071

[B26] Driesse MJ Vincent AJPE Smitt PS Kros JM Hoogerbrugge PM Avezaat CJJ Intracerebral injection of adenovirus harboring the HSVtk gene combined with ganciclovir administration: toxicity study in nonhuman primates Gene Ther 1998 5 1122 1129 10.1038/sj.gt.3300695 10326036

[B27] Driesse MJ Esandi MC Kros JM Avezaat CJJ Vecht C Zurcher C Intra-CSF administered recombinant adenovirus causes an immune response-mediated toxicity Gene Ther 2000 7 1401 1409 10.1038/sj.gt.3301250 10981667

[B28] Horst M Brouwer E Verwijnen S Rodijk M De JM Hoeben R Targeting malignant gliomas with a glial fibrillary acidic protein (GFAP)-selective oncolytic adenovirus J Gene Med 2007 9 1071 1079 10.1002/jgm.1110 17902184

[B29] Sillevis Smitt P Driesse M Wolbers J Kros M Avezaat C Treatment of relapsed malignant glioma with an adenoviral vector containing the herpes simplex thymidine kinase gene followed by ganciclovir Mol Ther 2003 7 851 858 10.1016/S1525-0016(03)00100-X 12788659

[B30] Vincent AJ Esandi MC Avezaat CJ Vecht CJ Sillevis Smitt P van Bekkum DW Preclinical testing of recombinant adenoviral herpes simplex virus-thymidine kinase gene therapy for central nervous system malignancies Neurosurgery 1997 41 451 452 10.1097/00006123-199708000-00023 9257313

