# Supplementary figures and images for: Truncating the i-leader open reading frame enhances release of human adenovirus type 5 in glioma cells
Source: Virol J. 2011 Apr 11;8:162. doi: 10.1186/1743-422X-8-162 (PMC3090740; doi:10.1186/1743-422X-8-162)

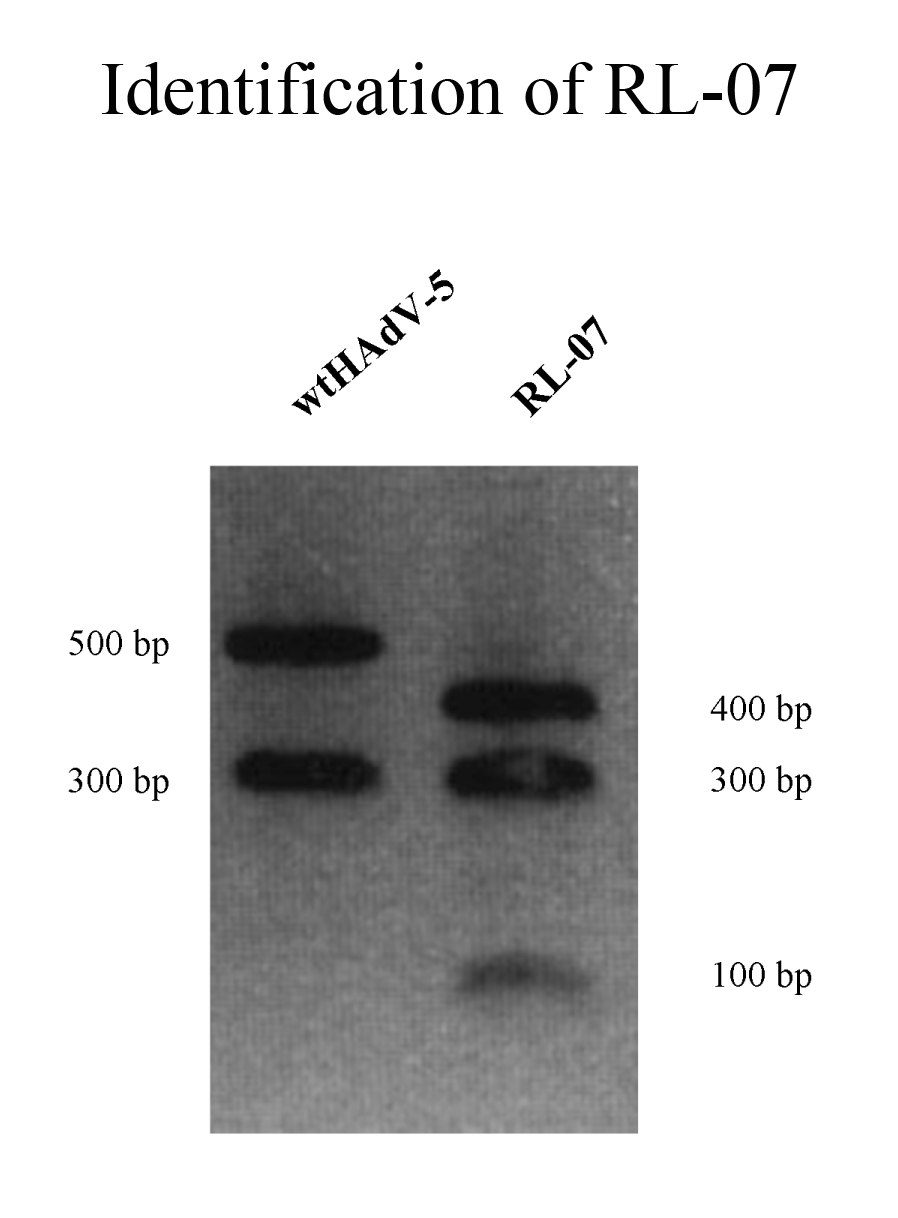

Supplement: Additional file 1 — Identification RL-07. Identification of RL-07 by PCR analyses. PCR analyses were performed on small freeze-thaw-lysate samples of the viral batches. Samples were heat inactivated and treated with proteinase K prior to PCR analysis. The i-leader region was amplified with the following primer set: Fwd primer 5'-AGACGCTCGGTGCGAGGATGCG; Rev primer 3'-GTCGTCTTCACGCAGAGGCGC. The PCR product was purified according to the SureClean protocol (Bioline, London, UK) and the product was digested using XhoI and loaded on 2% agarose gel. The picture represents the photo-negative image. [file 1743-422X-8-162-S1.TIFF]

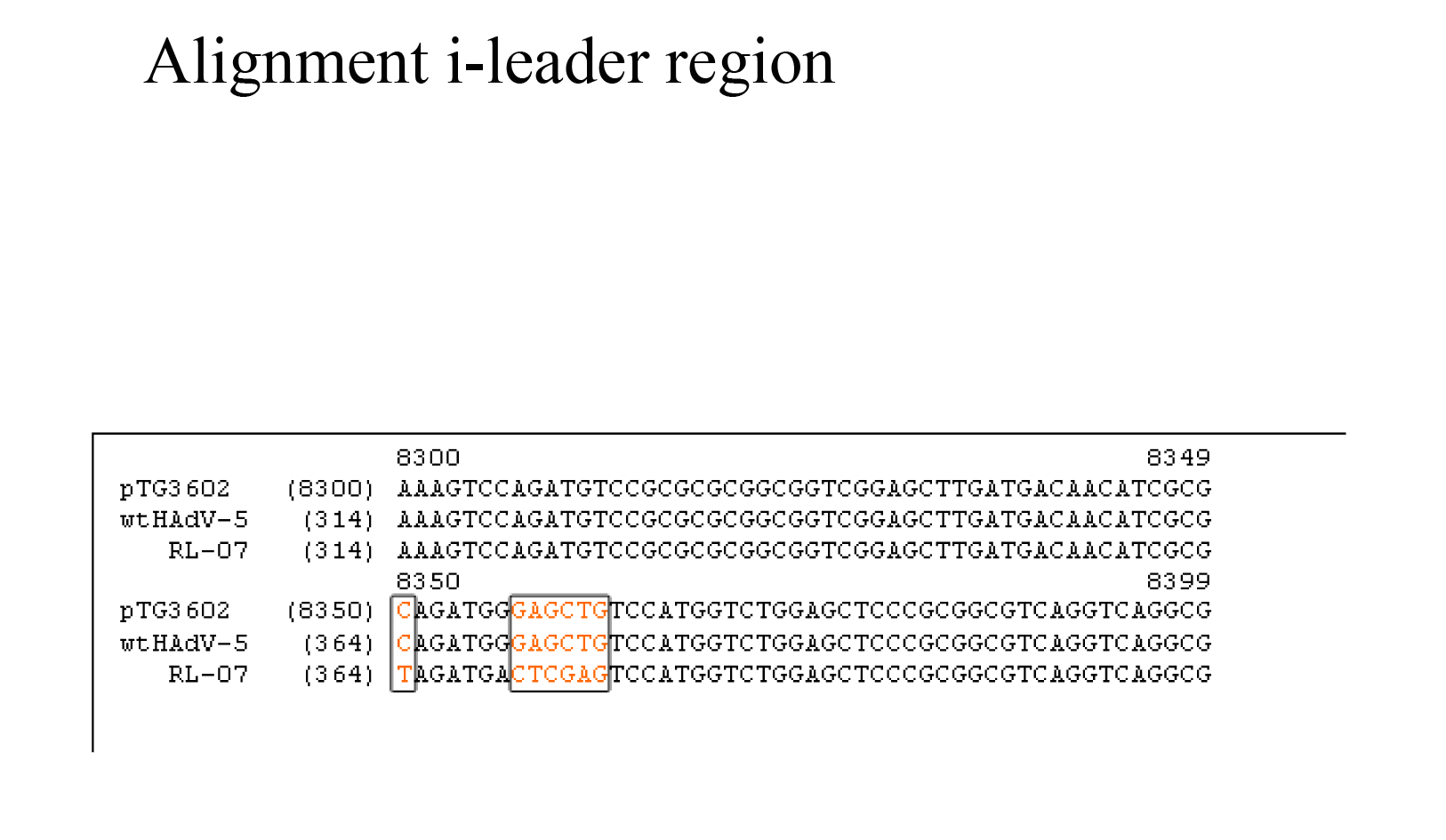

Supplement: Additional file 2 — Alignment i-leader region. Sequencing analyses were performed on PCR products of freeze-thaw samples of virus batches (described by identification RL-07). PCR products were first cleaned with Sureclean (Bioline, London, UK), according to the manual, before direct sequencing. Sequencing was performed at the Leiden Genome Technology Center (LGTC, Leiden, The Netherlands). The sequences of nucleotide 8300-8400 of the parental plasmid pTG3602 and the sequences of the HAdV-5 and RL-07 viruses are represented. The C8350T changes, as well as the XhoI site created in the RL-07 virus, are boxed. [file 1743-422X-8-162-S2.TIFF]
